# *In vitro* germ cell induction from fertile and infertile monozygotic twin research participants

**DOI:** 10.1016/j.xcrm.2022.100782

**Published:** 2022-10-18

**Authors:** Erica C. Pandolfi, Fei-Man Hsu, Mark Duhon, Yi Zheng, Sierra Goldsmith, Jianping Fu, Sherman J. Silber, Amander T. Clark

**Affiliations:** 1 Department of Molecular, Cell and Developmental Biology, University of California, Los Angeles, Los Angeles, CA, USA; 2 Eli and Edythe Broad Center of Regenerative Medicine and Stem Cell Research, University of California, Los Angeles, Los Angeles, CA, USA; 3 Molecular Biology Institute, University of California, Los Angeles, Los Angeles, CA, USA; 4 Department of Mechanical Engineering, University of Michigan, Ann Arbor, MI, USA; 5 Infertility Center of St. Louis, St. Luke’s Hospital, St. Louis, MO, USA; 6 Department of Biomedical Engineering, University of Michigan, Ann Arbor, MI, USA; 7 Department of Cell & Developmental Biology, University of Michigan Medical School, Ann Arbor, MI, USA

**Keywords:** hiPSCs, germ cells, twins, discordant POI, embryo model, amnion cells

## Abstract

Human induced pluripotent stem cells (hiPSCs) enable reproductive diseases to be studied when the reproductive health of the participant is known. In this study, monozygotic (MZ) monoamniotic (MA) twins discordant for primary ovarian insufficiency (POI) consent to research to address the hypothesis that discordant POI is due to a shared primordial germ cell (PGC) progenitor pool. If this is the case, reprogramming the twin’s skin cells to hiPSCs is expected to restore equivalent germ cell competency to the twins hiPSCs. Following reprogramming, the infertile MA twin's cells are capable of generating human PGC-like cells (hPGCLCs) and amniotic sac-like structures equivalent to her fertile twin sister. Using these hiPSCs together with genome sequencing, our data suggest that POI in the infertile twin is not due to a genetic barrier to amnion or germ cell formation and support the hypothesis that during gestation, amniotic PGCs are likely disproportionately allocated to the fertile twin with embryo splitting.

## Highlights

• Monozygotic (MZ) monoamniotic (MA) twins with discordant POI are evaluated here
• Each twin’s hiPSCs lines make separate amniotic sacs with germ cells *in vitro*
• Competency to generate germ cells is equivalent between twins with discordant POI

## Graphical abstract

**Figure:**
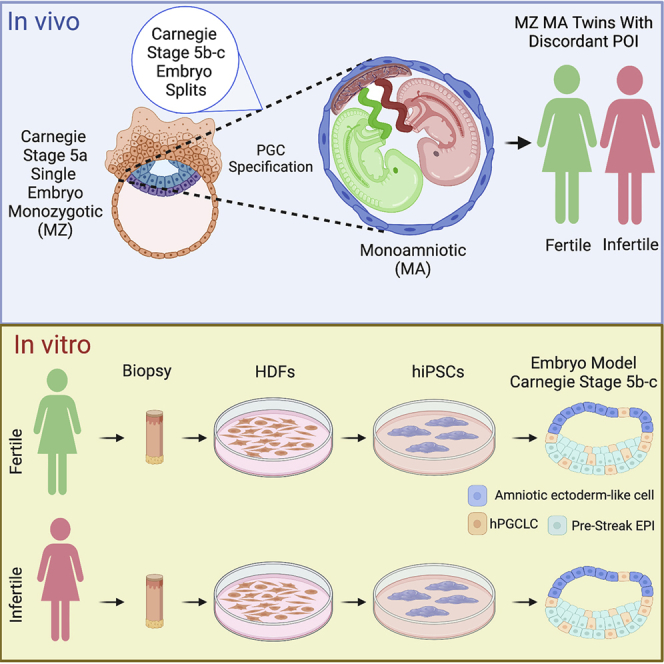

## Teaser

Pandolfi et al. use hiPSCs to evaluate germ cell and amnion formation in MZ MA twins discordant for primary ovarian insufficiency (POI). Results suggest a barrier to germ cell formation such that the infertile twin is unable to generate sufficient germ cells to overcome POI as young adult, which reprogramming to hiPSCs can reverse.

## Introduction

The earliest human embryonic stem cell (hESC) lines were derived from isolated inner cell masses of human blastocysts consented to research.^1^ Almost a decade later, human induced pluripotent stem cell (hiPSC) lines were derived.^2^, ^3^, ^4^ Since then, thousands of patient-specific hiPSC lines from “healthy” or “disease” research participants have been generated, catalyzing stem cell science in exciting and collaborative ways.^5^, ^6^, ^7^ Critical to the use of pluripotent stem cells is the informed consent process and a framework for scientific and ethical oversight.^8^ Certain areas of basic science research with pluripotent stem cells are considered sensitive, such as reproductive science research and the differentiation of germ cells and gametes, a technique called *in vitro* gametogenesis (IVG).

Bioethicists consider the differentiation of germ cells from pluripotent stem cells without the intent to make embryos ethically similar to differentiating somatic cells.^8^ However, patient and community perspectives in this area are starting to emerge, with a recent study revealing that research participants deem gonadal organoids (containing germ cells) as morally distinct from other types of organoid research.^9^ Balancing patient perspectives with the current state of the science, the 2021 update to the International Society for Stem Cell Research (ISSCR) “Guidelines for Stem Cell Research and Clinical Translation” recommend that differentiation of primordial germ cells (PGCs) from pluripotent stem cells in non-integrated stem cell-based embryo models be reportable to a specialized scientific and ethics oversight review process but not normally subject to further review.^10^^,^^11^ In contrast, experiments involving use of IVG-derived gametes to generate embryos for research purposes should be subject to a specialized review process.^10^^,^^11^

Deriving disease-specific hiPSC lines for research tends to focus on devastating lethal diseases. Absent from the disease lists in most iPSC repositories is infertility. Infertility is a disease of the reproductive system defined as a failure to achieve a pregnancy after 12 months of trying.^12^ Current estimates indicate that infertility affects between 48 million couples and 186 million individuals globally.^13^^,^^14^ Causes of infertility are varied and diagnosed in all genders; however, a failure to specify PGCs will cause certain infertility given that gametes (which originate from PGCs) are the only cells in the body capable of fertilization. In the adult ovary, a lack of germ cells causes ovarian failure, also referred to as primary ovarian insufficiency (POI). A lack of germ cells in a pre-pubescent child’s ovaries will result in failure to transition through puberty and POI. POI is not a rare disease, with 1% of women experiencing ovarian failure before the age of 40.^15^ A baby assigned female at birth does not generate new oocytes after birth,^16^ which is why establishment of an appropriate number of germ cells and oocytes during the pre-natal window is a critical determinant of whether a person will experience POI.

POI is more common among twins than the general population, with monozygotic (MZ) twins affected at 3- to 5-fold higher rates than un-related individuals.^17^, ^18^, ^19^ MZ, monochorionic (MC), monoamniotic (MA) twins (also called MA twins) are a rare subset of twins occurring in 1 in every 100 sets of MZ twin births.^20^^,^^21^ MA twin pairs are especially useful for investigating potential epigenetic causes of discordant diseases, including POI, as they are genetically similar, and the twins shared the same *in utero* environment prior to birth.^21^^,^^22^ An MA twin pair splits from a single MZ embryo between days 8–13 post-fertilization (pf) or at Carnegie stage (CS) 5b-c and thus share a placenta, an amnion, and a chorion. Importantly, MA twin splitting occurs around the time PGC specification, which begins at CS5b in primates,^23^^,^^24^ suggesting that these twins may also share a PGC progenitor pool located in the amnion. Given this, the hypothesis to be addressed is that reprogramming somatic cells from MA twin pairs discordant for POI into hiPSCs could reset the epigenome of the affected twin such that she is able to produce PGC-like cells (human PGC-like cells [hPGCLCs]) and amnion equivalent to her fertile sister.

## Results

### Human subject selection

**Figure 1:**
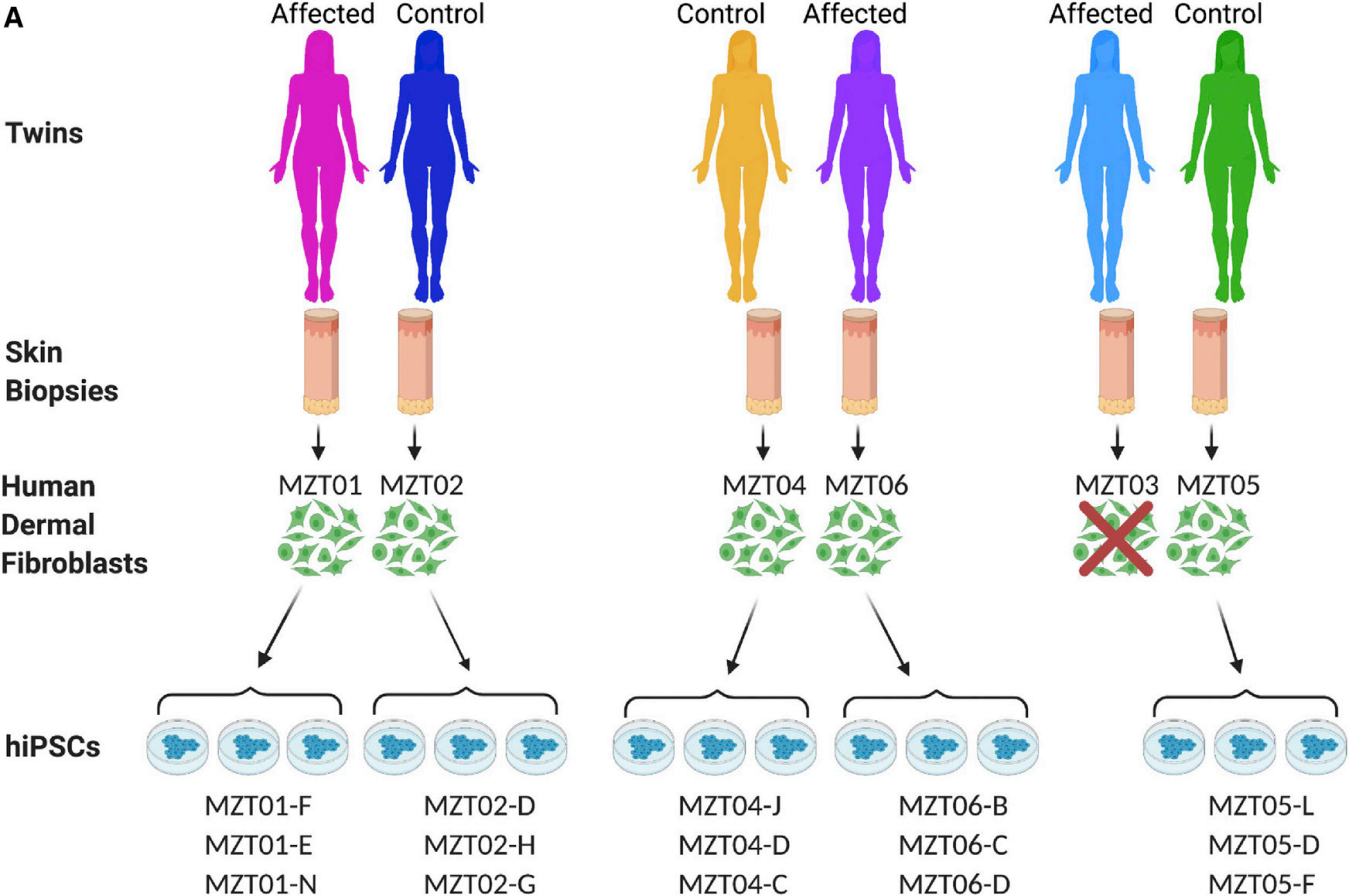
Derivation of HDFs and hiPSCs from twin pairs with discordant POI (A) Diagram depicting relationship of research participants and their HDFs and hiPSC sublines. Sublines are used as biological replicates for each participant. Figure created with BioRender.com.

Participants were consented into this research study from a cohort of MZ twins discordant for POI who were treated at the Infertility Center of St. Louis (MO, USA). Three sets of MZ twins (two of which are known to be MA) donated skin punch biopsies to this study (Figure 1A). MZT01 was diagnosed with early-onset menopause due to POI at age 25. Her twin sister, MZT02, a woman with normal fertility during her reproductive years, successfully gave birth without intervention. MZT01 underwent ovarian transplant surgery where she received an ovary from her fertile twin sister MZT02 at the Infertility Center of St. Louis, and following this procedure, she subsequently gave birth to two children (Table S1). The second twin pair consists of MZT03, a woman diagnosed with early-onset menopause due to POI at age 31, and her twin sister, MZT05, a woman with normal fertility during her reproductive years who successfully gave birth without intervention. MZT03 underwent ovarian transplant surgery, receiving an ovary from her fertile twin sister MZT05 at the Infertility Center of St. Louis, and she subsequently gave birth to one child. MZT03 was also diagnosed with leukemia and underwent radiation and chemotherapy treatment prior to consenting to this study. The final twin pair consists of MZT04, a woman with normal fertility during her reproductive years, and her twin sister, MZT06, a woman with early-onset menopause due to POI at age 22. MZT06 received an ovary transplant from her twin sister MZT04 and subsequently gave birth to three children.^20^

Following informed consent, the women donated a skin punch biopsy, and human dermal fibroblasts (HDFs) were successfully isolated. All HDF samples were karyotypically normal except MZT03, which did not yield karyotypically normal HDFs despite two skin biopsy donations. Therefore, this sample was not used in further experiments. The remaining five HDF samples were reprogrammed into hiPSCs, with three colonies picked for further analysis resulting in karyotypically normal, self-renewing pluripotent sublines that are genetically identical to their parental HDFs as confirmed by short tandem repeat (STR) analysis^25^, ^26^, ^27^, ^28^ (Figure 1A). Three hiPSC sublines from each research participant were used as biological replicates in the following experiments.

### Whole-genome sequencing (WGS) suggests no causative POI mutations in the twins

As STR does not evaluate POI candidate genes, we first sought to expand the genome analysis to include WGS (20 samples in total) (Table S2). Jaccard indices^29^ were used to calculate the relatedness of HDF samples, confirming high similarity between twin pairs MZT04/MZT06 and MZT01/MZT02 (values close to 1.0) as well as their unrelatedness to MZT05 (Figure S1A). Next, we queried the sequence of 22 genes previously associated with POI (Table S3). No genomic discrepancies between twins were observed in the protein-coding regions of these 22 genes; however, single-nucleotide variants (SNVs) were identified in an intron of *FSHR* and *PMM2* in MZT01 and MZT06 (POI participant) but not their fertile twin sisters MZT02 or MZT04. These SNVs were identified as common SNPs in the human population and have not been reported as pathological in dbSNP.^30^

The reprogramming process is known to introduce genomic alterations.^31^, ^32^, ^33^, ^34^, ^35^, ^36^, ^37^ Given that multiple hiPSC sublines were derived from each HDF sample, we next sought to assess the number of genomic changes acquired by each hiPSC subline. We applied two approaches (GATK and Strelka),^38^, ^39^, ^40^ standard bioinformatics approaches for analyzing cancer genomes, treating the hiPSCs as “tumor” and the matched HDFs as “normal.” To generate a stringent call set, we retained only genomic changes detected by both methods and an allelic frequency greater than 10%.^41^^,^^42^ Consistent with previous reports,^35^ we found that the number of mutations varied between sublines following reprogramming (Figure S1B; Table S2), with the average number of acquired mutations being 1.2 mutations/Mb (Figure S1C). The majority of mutations correspond to SNVs of the C > T or T > C type. Four hiPSC sublines (MZT02-G, MZT02-H, MZT01-N, and MZT04-J) acquired >10,000 mutations (Figure S1D).

### Induced reprogramming restores germ cell competency to the infertile twin

**Figure 2:**
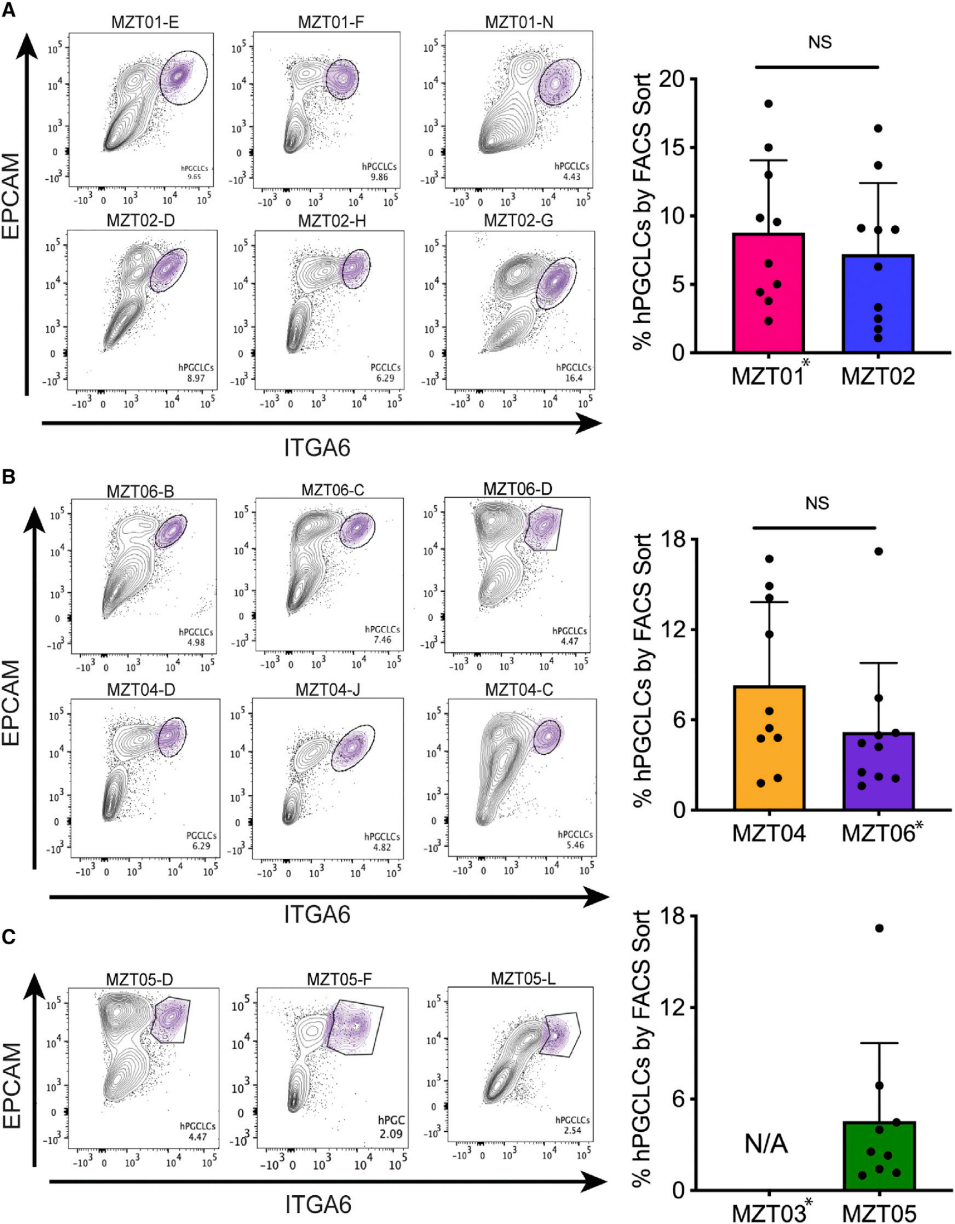
Percentage of hPGCLCs differentiated from hiPSCs The hiPSC sublines for each participant (n = 3 biological replicates) were each differentiated a minimum of three times. (A) Fluorescence-activated cell sorting (FACS) plots and quantification of hPGCLC percentages at day 4 of aggregate differentiation from twin pair MZT01 and MZT02 (t test, n = 10, p = 0.36). (B) Twin pair MZT04 and MZT06 (t test, n = 10, p = 0.73). (C) MZT05. The hPGCLC population is identified as double-positive for EPCAM and ITGA6 (circle). Data are represented as mean ± SEM with each circle on the graph, indicating the hPGCLC population. Statistical significance was calculated using a t test.

In order to assess germ cell differentiation in each hiPSC subline, we induced the hiPSCs into incipient mesoderm-like cells (iMeLCs)^43^ followed by differentiation as three-dimensional (3D) aggregates in round-bottom low-adhesion 96-well plates in media containing BMP4 and other cytokines.^43^ The percentage of hPGCLCs in the aggregates was quantified using fluorescence-activated cell sorting (FACS) at day 4 of aggregate differentiation with integrin alpha 6 (ITGA6) and epithelial cell adhesion molecule (EPCAM), two cell-surface markers of hPGCs (Figures 2A–2C).^43^^,^^44^ There was no significant difference in hPGCLC percentage when comparing MZT01 with MZT02 or MZT04 with MZT06. Furthermore, hiPSCs derived from MZT05, the fertile twin sister of MZT03, also produced comparable percentages of hPGCLCs to the other participants. Secondary analysis comparing sublines derived from the same participant revealed no significant difference (Figure S2A), indicating that the especially high numbers of DNA mutations in MZT02-G, MZT02-H, MZT01-N, and MZT04-J did not alter hPGCLC competency. Immunofluorescence (IF) analyses for PGC markers SOX17, PRDM1, and TFAP2C^45^, ^46^, ^47^, ^48^ (Figures S2B–S2D) further verified hPGCLC identity at day 4.

### Germline and somatic gene expression is equivalent between the twins

Next, we performed single-cell RNA sequencing (scRNA-seq) using 10X Genomics to evaluate gene expression profiles of hPGCLCs and somatic cells in all hiPSC sublines (Table S4). Single cells from each subline were collected at day 4 of aggregate differentiation consistent with previous studies.^23^^,^^44^^,^^49^, ^50^, ^51^, ^52^

**Figure 3:**
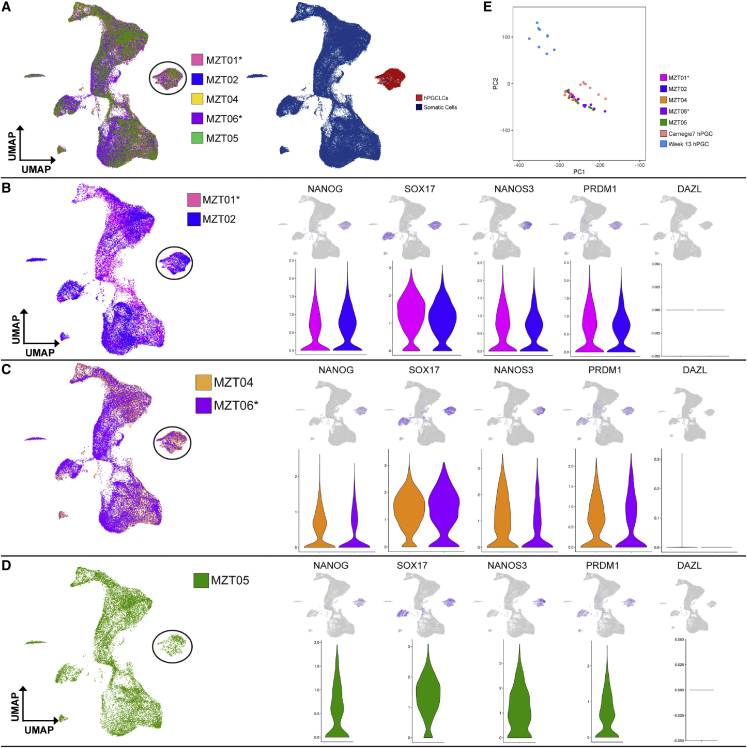
Differentiation of each MZ twin’s hiPSCs yields hPGCLCs with similar identity (A) The hiPSC sublines from each participant (n = 3 biological replicates) were analyzed by 10X Genomics to evaluate germline and somatic cell identity at day 4 of aggregate differentiation. *In vivo* hPGCs from CS7 human embryos^53^ were used to stage hPGCLC development. (B–D) Germ cell gene expression was evaluated in the hPGCLCs from (B) twins MZT01 and MZT02, (C) twins MZT04 and MZT06, and (D) MZT05. The hPGCLC population analyzed on the right is indicated by the circled population of cells on the left. (E) Principle component analysis of the hPGCLC populations in this study with CS7 hPGCs and hPGCs from week 13 fetal gonads. The ∗ indicates the affected twin.

The hPGCLC population within the aggregate was defined as a clearly separated cluster expressing hPGC markers *NANOG*, *SOX17*, *NANOS3*, and *PRDM1* (Figures 3A–3C). Absence of *DAZL* (Figures 3B and 3C) indicates that the hPGCLCs are in an early stage and have not undergone determination to create committed hPGCs (also called late-stage PGCs or gonocytes). In a principle-component analysis (PCA), all hPGCLCs clustered near hPGCs from CS7 human embryos^53^ (Figure 3E), reinforcing the notion that hPGCLCs in the current study are equivalent to early hPGCs rather than committed hPGCs in the embryonic gonad. Furthermore, the hPGCLCs from each twin clustered together regardless of their fertility states (Figure 3A), with no statistically significant difference in hPGCLC gene expression between the twin pairs, including expression levels of key hPGCLC/hPGC genes (Figures 3B and 3C). These data indicate that the stage and transcriptional identity of hPGCLCs differentiated from hiPSC lines derived from all research participants are very similar regardless of fertility diagnosis and correspond to hPGCs *in vivo* at ∼CS7.

Transcriptome analysis of somatic cells in the aggregate revealed expression of somatic lineage markers (Figures S3A–S3C). This includes rare cells expressing *TBXT* and *MIXL1*, which likely mark primitive streak-like cells. *FOXA2*+ cells, which likely mark endoderm, as well as a large fraction of HAND1+ cells, of which a subset express *TFAP2A*, *IGFBP5*, and *GABRP*, recently identified markers of amnionic ectoderm.^54^ These cells, which we putatively call “amnion-like cells,” clustered with the amnion-like cells identified by Zheng et al.^55^ (Figure S3D), suggesting that 3D aggregate differentiation of hiPSCs in the presence of BMP4, LIF, and ROCKi generates cells by day 4 that resemble amniotic ectoderm.

### Each twin is competent to generate her own amniotic sac containing hPGCLCs

**Figure 4:**
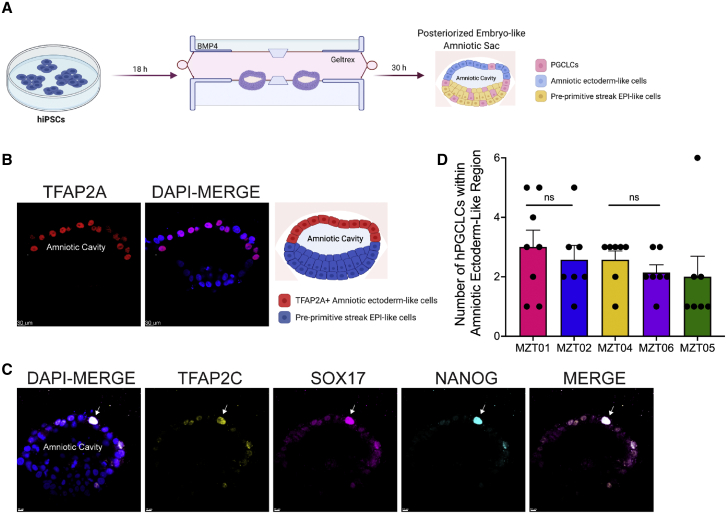
Induction of hPGCLCs in an embryo model of the amniotic sac (A) Diagram of the embryo model. BMP4 was added 18 h after loading hiPSCs into the device. Thirty h after adding BMP4, amniotic sac-like embryo models develop, each containing an amniotic cavity, an amniotic ectoderm-like cell layer, pre-primitive streak epiblast (EPI)-like cells, and hPGCLCs (right). (B) The amniotic ectoderm-like cell layer is TFAP2A+ (n = 12 modeled embryos per participant were evaluated). Scale bar: 30 μm. (C) Representative images of hPGCLCs (triple positive for TFAP2C, NANOG, and SOX17) in the amniotic ectoderm-like cell layer (shown is MZT05-D). Arrows indicate hPGCLCs. (D) The number of specified hPGCLCs in the amniotic ectoderm-like cell layer was quantified from n = 8 embryo models from each participant’s hiPSCs (MZT01 versus MZT02, p = 0.58; MZT04 versus MZT06, p = 0.30). Scale bar: 10 μm. Data are represented as mean ± SEM. Figure created with BioRender.com. Statistical significance was calculated using t test.

The gestational history of two MZ twin pairs in this study indicate that, while *in utero*, MZT01/MZT02 and MZT04/MZT06 shared an amnion and a chorion. Given that hPGCs in primates *in vivo* are specified around the time of amnion formation,^24^ we next asked whether each twin can generate her own amniotic sac-like structure using a non-integrated embryo model. To achieve this, we used a microfluidics approach to generate posterior embryonic-like sacs,^55^ which recapitulates *in vivo* 3D amniotic sac tissue architecture and spatiotemporal lineage development reminiscent of those in the early post-implantation human embryos at the time of PGC specification (Figure 4A). To create this embryo model, hiPSCs from each twin were injected into microfluidic devices, and 30 h after exposure to BMP4, individual embryo models formed, containing a squamous amniotic ectoderm-like cell layer at the pole directly opposed to BMP4, columnar epiblast-like cells at the opposite pole, and hPGCLCs^55^ (Figure 4A). The presence of amniotic ectoderm-like cells within the embryo model were confirmed using IF for TFAP2A (Figure 4B).^55^ The emergence of hPGCLCs was evaluated using IF for NANOG, TFAP2C, and SOX17 with an average of ∼2–3 hPGCLCs identified as NANOG+ TFAP2C+ SOX17+ triple-positive cells, in the amniotic ectoderm-like cell layer (Figures 4C, 4D, S4A, and S4B). There was no statistical difference in the number of hPGCLCs in the amniotic ectoderm-like cell layer generated from each twin (Figure 4D). We also identified hPGCLCs in the pre-primitive streak EPI (Figures S4A and S4B) as previously reported,^55^ consistent with the porcine model of PGC specification.^56^

## Discussion

In this study, we consented MZ twins with discordant POI. Two sets of unrelated twins in this study had gestational histories indicating that each pregnancy involved a twin pair gestating within a single amnion.^17^^,^^18^^,^^20^ Given recent evidence that specified primate PGCs are situated in the dorsal amnion at CS 5b prior to gastrulation, a possible scenario is that MA twins also share the amniotic PGC progenitor pool. Therefore, it could be hypothesized that at the time of embryo splitting, shared amniotic PGC progenitors disproportionally allocate to the fertile twin. Disproportionate allocation of cells during embryo cleavage is a phenomenon supported by the variable allelic frequency range observed between twin pairs after splitting.^57^ WGS was performed to exclude the possibility that the infertile twin had disproportionately acquired cells with mutations in POI-associated genes that could also explain the discordant POI phenotype. Instead, our data support the hypothesis that at the time of embryo splitting, an epigenetic barrier to PGC specification was likely established, and the infertile twin was unable to generate a sufficient cohort of additional PGCs in order to overcome POI as young adult.

Given that MA twins share an amniotic sac *in utero,* we used a non-integrated human embryo model to evaluate amniotic-like sac formation from each participant’s cells.^55^ Using hiPSCs, we demonstrated that all research participants are competent to generate their own amniotic sac-like structures containing equivalent numbers of hPGCLCs. Thus, the single amnion in these MA twins is likely due to the timing of embryo splitting and not a genetic barrier to amnion formation. This analysis does not prove that hPGCs *in vivo* are specified exclusively in the amnion. Rather, our data indicate that specified hPGCs are consistently and reliably identified in the amniotic ectoderm-like cell layer *in vitro*, and we propose it is likely the extra-embryonic pool of PGCs that are disproportionally allocated to the fertile sister in the MA twins discordant for POI.

Prior to this study, we reported that the HDFs and hiPSCs used for this study were karyotypically normal.^25^, ^26^, ^27^, ^28^ WGS in the current study revealed variable subkaryotypic changes in each twin’s hiPSCs relative to the original HDFs. These genomic changes occurred with reprogramming and corresponded to as few as 276 acquired SNVs in MZT01-F to as high as 332,530 acquired SNVs in MZT02-G. Our data corroborate previously published studies demonstrating that large differences in the number and type of genomic changes can be identified when comparing different hiPSC subclones derived from the same individual.^35^ Similar to other studies, the hiPSC sublines generated here also contained a higher fraction of T > C or C > T SNVs, an occurrence associated with high rates of hydrolytic deamination of cytosine bases.^58^ Our study revealed that the hiPSC sublines containing the highest numbers of mutations do not show any difference in hPGCLC differentiation potential. Therefore, a high frequency of genomic mutations acquired with reprogramming does not serve as a barrier to hPGCLC differentiation *in vitro* or as selectable criteria for excluding hiPSC sublines from downstream studies. Genomic alterations in HDF-derived hiPSCs advances our understanding of the genomic impact of reprogramming and culturing on human cells, providing a reference point for discussions of tolerable mutation level when considering the safety of IVG for potential future reproductive purposes. Critically, in the current study, all hiPSC sublines had a higher number of genomic changes than would be anticipated during human germline development *in vivo.*^59^, ^60^, ^61^ We believe that this concern must be addressed before using gametes generated by IVG for reproductive purposes.

The work presented here shows that when epigenetic reprogramming is used to create hiPSCs from MA twins discordant for POI, germ cell differentiation from the resulting hiPSC sublines is equivalent regardless of whether aggregate or embryo models are used. Our ability to derive hPGCLCs from these hiPSC sublines with a similar transcriptome to hPGCs from CS7 embryos, suggests that these hPGCLCs may have the potential to differentiate further into oocytes under appropriate culture conditions.

### Limitations of the study

This work used hiPSCs to model developmental events occurring at the time of amnion formation and MA twinning, which could be used to explain the high incidence of discordant POI in MA twins. Although our work demonstrates that hiPSCs derived from each MA twin pair have equivalent capacity to induce germ cells and develop amniotic sacs, this study does not evaluate post-natal or adult stages of germ cell and follicle formation, where discordant POI phenotypes could also arise. Unfortunately, the technologies for differentiating follicles from hiPSCs capable of folliculogenesis do not currently exist; however, once these technologies are established, future studies could address this. In addition, given the diversity in POI etiologies, extrapolation of these findings to other MA twins outside of this study is unclear.

## STAR★Methods

### Key resources table

**Table:** 

REAGENT or RESOURCE	SOURCE	IDENTIFIER
**Antibodies**		

anti-TFAP2C	Santa Cruz	AB_2286995
anti-TFAP2C	Santa Cruz	AB_667770
anti-PRDM1	Cell Signaling	AB_2169699
anti-SOX17	Neuromics	AB_2195648
anti-NANOG	Abcam	AB_10863442
anti-TFAP2A	Santa Cruz	AB_667767
donkey anti-mouse IgG AF594	Thermofisher	AB_141633
donkey anti-rabbit IgG AF488	Thermofisher	AB_2535792
donkey anti-goat IgG AF647	Thermofisher	AB_141844
donkey anti-mouse IgG_2b_ AF594	Thermofisher	AB_2535781
goat anti-mouse IgG_2a_ AF488	Thermofisher	AB_2535771
anti-ITGA6 AF421	BioLegend	313607
anti-EPCAM AF488	BioLegend	324210

**Deposited data**		

hPGCLC Aggregate scRNA-seq from all hiPSC sublines	GEO	GSE181205
WGS HDFs and hiPSC sublines	NCBI BioProject	PRJNA759332.

**Experimental models: Cell lines**		

MZT04-D	https://hpscreg.eu/cell-line/UCLAi001-A	UCLAi001-A
MZT04-J	https://hpscreg.eu/cell-line/UCLAi001-B	UCLAi001-B
MZT04-C	https://hpscreg.eu/cell-line/UCLAi001-C	UCLAi001-C
MZT01-E	https://hpscreg.eu/cell-line/UCLAi002-A	UCLAi002-A
MZT01-F	https://hpscreg.eu/cell-line/UCLAi002-B	UCLAi002-B
MZT01-N	https://hpscreg.eu/cell-line/UCLAi002-C	UCLAi002-C
MZT02-D	https://hpscreg.eu/cell-line/UCLAi003-A	UCLAi003-A
MZT02-G	https://hpscreg.eu/cell-line/UCLAi003-B	UCLAi003-B
MZT02-H	https://hpscreg.eu/cell-line/UCLAi003-C	UCLAi003-C
MZT06-B	https://hpscreg.eu/cell-line/UCLAi004-A	UCLAi004-A
MZT06-C	https://hpscreg.eu/cell-line/UCLAi004-B	UCLAi004-B
MZT06-D	https://hpscreg.eu/cell-line/UCLAi004-C	UCLAi004-C
MZT05-D	https://hpscreg.eu/cell-line/UCLAi005-A	UCLAi005-A
MZT05-F	https://hpscreg.eu/cell-line/UCLAi005-B	UCLAi005-B
MZT05-L	https://hpscreg.eu/cell-line/UCLAi005-C	UCLAi005-C
HDF MZT01		Pandolfi et al.,^28^
HDF MZT02		Pandolfi et al.,^28^
HDF MZT04		Pandolfi et al.,^27^
HDF MZT05		Pandolfi et al.,^26^
HDF MZT06		Pandolfi et al.,^25^

### Resource availability

#### Lead contact

Further information and requests for resources and reagents should be directed to and will be fulfilled by the Lead Contact, Amander Clark (clarka@ucla.edu).

#### Materials availability

hiPSC lines used in this study^25^, ^26^, ^27^, ^28^ were generated at UCLA and are available upon request to the Lead Author with MTA and appropriate institutional approvals for working with human induced pluripotent stem cells.

### Experimental model and subject details

#### Human dermal fibroblasts (HDFs)

The karyotypically normal human dermal fibroblast (HDF) samples used in this study were previously published.^25^, ^26^, ^27^, ^28^ The names of the HDFs are; MZT01, MZT02, MZT04, MZT05 and MZT06. These fibroblasts originated from a skin punch biopsy donated from the five women who at the time of biopsy were aged 39–53 as indicated in Table S1. MZT01 and MZT06 were previously diagnosed with POI, their respective twin sisters MZT02 and MZT04, had normal fertility during their reproductive years. Upon thawing, HDFs are cultured at 37°C, 5.0% CO_2_ on tissue culture treated plates coated with 0.1% gelatin (Sigma) in media consisting of 15% Fetal Bovine Serum; FBS (GE Healthcare), 1% Non-Essential Amino Acids (Invitrogen), 1% Glutamax (GibcoTM), 1% Penicillin-Streptomycin-Glutamine (Gibco) and Primocin (Invogen). Consent to a skin biopsy, generation of HDFs, generation of hiPSCs and differentiation of hiPSCs was approved and annually reviewed by the UCLA Institutional Review Board (IRB #16-001176) together with additional approval and annual review by the UCLA Human Pluripotent Stem Cell Research and Oversight (hPSCRO) Committee (hPSCRO #2016-003). Mycoplasma was regularly tested before banking using the MycoAlert kit from Lonza Catalog #LT07-318.

#### Human induced pluripotent stem cell (hiPSC) lines

For each HDF sample, we used n = 3 previously published hiPSC sublines,^25^, ^26^, ^27^, ^28^ which were generated under the same consent and approval process as the HDFs described above (IRB, 16-001176 and hPSCRO# 20016-003). All hiPSC subline cells were cultured at 37°C, 5.0% CO_2_ on a feeder layer of mitomycin C-treated murine embryonic fibroblasts (MEFs) in pluripotent stem cell media (DMEM/F-12) (Life Technologies), 20% KSR (Life Technologies), 10 ng/mL bFGF (R&D Systems), 1% nonessential amino acids (Life Technologies), 2 mM L-glutamine (Life Technologies), Primocin™ (Invivogen), and 0.1 mM β-mercaptoethanol (Sigma). Media was changed daily and colonies were passaged with collagenase type IV (ThermoFisher, 17104019) every 7 days. For some experiments, cells were also maintained in feeder-free conditions on Matrigel (Fisher Scientific, 08-774-552) in mTeSR (STEMCELL Technologies 85850) at 37°C, 5.0% CO_2_. For mTeSR conditions, media was changed daily and colonies were passaged with ReLeSr (STEMCELL Technologies 05873) every 5 days. Mycoplasma was regularly tested using MycoAlert kit from Lonza Catalog #LT07-318.

### Method details

#### Induction of hPGCLCs in aggregates

To induce hiPSCs into hPGCLCs, the hiPSCs cultured for 7-days on mitomycin C-treated MEFs were trypsinized for 5 minutes (0.05% trypsin, Life Technologies) before resuspending in trypsin inhibitor (Life Technologies) to quench the trypsin. The MEFs weree depleted from the cell suspension by plating the cells onto tissue culture dishes, two times for 5 min each. The MEF-depleted cell suspension was then collected from the second plate, pelleted at 1,000 rpm using a centrifuge and resuspended in iMeLC media composed of (GMEM) (Life Technologies) containing 15% KSR (Life Technologies), 0.1 mM nonessential amino acids (Life Technologies), penicillin/streptomycin/L-glutamine (Life Technologies), Primocin™ (Invivogen), 0.1 mM β-mercaptoethanol (Sigma), sodium pyruvate (Life Technologies), activin A (PeproTech), CHIR99021 (Stemgent) and Y-27632 (Stemgent). After re-suspending in iMELC media, the cell suspension was filtered through a 40 μm cell strainer (Falcon) and plated at a density of 2.0 × 10^5^ cells per well of a human plasma fibronectin (Invitrogen)-coated 12-well plate. After 24 h of incubation at 37°C with 5.0% CO_2_, the cells are referred to as iMeLCs. At this point, the cells are harvested from the 12-well plate by incubating in 0.05% trypsin for 5 minutes (Life Technologies) before resuspending in aggregate media consisting of (GMEM) (Life Technologies), 15% KSR (Life Technologies), 0.1 mM nonessential amino acids (Life Technologies), penicillin/streptomycin/L-glutamine (Life Technologies), Primocin™ (Invivogen), 0.1 mM β-mercaptoethanol (Sigma), and sodium pyruvate (Life Technologies) containing 10 ng/mL human LIF (EMD Millipore), 200 ng/mL BMP4 (R&D Systems), 50 ng/mL EGF (Fisher Scientific) and 10 μM Y-27632 (Stemgent). The single cell suspension is then added to low adherence 96-well plates (Corning) at a density of 3.0 × 10^3^cells per well to make the aggregates. All experiments analyzing hPGCLC induction in the aggregates are performed on day 4 (D4) after generating aggregates in 96 well plates. This technique was first described by Sasaki et al.,^43^ with minor modifications adopted by Chen et al.^44^ The detailed methods described above use the modified protocol adopted by Chen et al.^44^

#### Analysis of hPGCLCs from aggregates using fluorescence-activated cell sorting (FACS)

Aggregates at day 4 of differentiation were dissociated using 0.05% trypsin. Cells were washed with MEF media and then re-suspended in FACS buffer. Dissociated cells were incubated with anti-ITGA6-BV421 (BioLegend 313624; 1:50) and EPCAM-488 (BioLegend 324210; 1:50) antibodies. Double positive cells were collected using an ARIA-H Fluorescence Activated Cell Sorter. Analysis of hPGCLC percentages was performed on n = 3 sublines (biological replicates) in 3–4 independent experiments (technical replicates) for a total of 10 replicates per participant. Cytometry analysis was performed using FlowJo™ version 10.

#### Immunofluorescence (IF) staining

Aggregates containing hPGCLCs were collected and then fixed in 4% paraformaldehyde (PFA) for one hour before embedding in histogel (Thermo Scientific, 22-110-678) followed by paraffin blocks. Paraffin blocks were sectioned at 5 μm onto glass slides prior to IF staining. IF staining began with de-paraffinizing the sections in xylene (Fisher Scientific, X3P-1GAL) followed by re-hydration in an ethanol series from 100% ethanol to 30% ethanol. For antigen retrieval, slides were heated to 95°C in Tris-EDTA solution (10 mM Tris Base, 1 mM EDTA solution, 0.05% Tween-20, pH 9.0) for 45 minutes. Sections were permeabilized (PBS, 0.05% Triton-100) for 10 minutes and then blocked in PBS containing 10% normal donkey serum for 30 minutes. The primary antibodies anti-TFAP2C (sc8977; 1:200, RRID: AB_2286995), anti-TFAP2C (sc12762; 1:200, RRID: AB_667770), anti-BLIMP1 (Cell Signaling C14A4; 1:100, RRID: AB_2169699), anti-SOX17 (GT15094; 1:100, RRID: AB_2195648), anti-BRACHYURY/T (Fisher Scientific AF2085; 1:200, RRID: AB_2200235), anti-NANOG (ab109250, 1:40, RRID: AB_10863442), anti-TFAP2A (sc-12726, 1:200, RRID: AB_667767) were incubated overnight at 4°C. Primary incubation was then followed by incubating the slides for 60 min at room temperature with their corresponding species-specific secondary antibodies: donkey anti-mouse IgG AF594 (A21203; 1:200, AB_141633), donkey anti-rabbit IgG AF488 (A21206; 1:500, AB_2535792), donkey anti-goat IgG AF647 (A21447; 1:200, AB_141844), donkey anti-mouse IgG_2b_ AF594 (A21145; 1:200, AB_2535781), goat anti-mouse IgG_2a_ AF488 (A21131; 1:200, RRID: AB_2535771). Mounting media (Prolong-gold anti-fade w/DAPI, Invitrogen) was added and slides were sealed before imaging.

#### Embryo model of the amniotic-sac

Microfluidic devices for generating non-integrated model embryos were create as described previously.^55^ Devices were created by pouring PDMS (1:10 weight ratio) (Sylgard, 761036) gel into molds and bonded to glass coverslips (Fisher Scientific, 50-189-9793) after curing. The Loading channel of devices was filled with geltrex (ThermoFisher, A1413302) diluted in E6 (Life Technologies, A1516401) (3:7.5, E6:geltrex). hiPSC lines adapted to feeder-free conditions in mTeSR (STEMCELL Technologies 85850) on Matrigel (Fisher Scientific, 08-774-552) were enzymatically digested with Trypsin (0.05%) for three minutes, quenched with Trypsin inhibitor (Life Technologies, 17075029), and resuspended in mTeSR at a concentration of 8 × 10ˆ5 cells/mL. The cell suspension was then added to the cell loading well at a volume of 10uL, and cells were allowed accumulated within the pockets for five minutes. Media was then added to the wells, and cells were incubated overnight at 37°C. 24 hours later, 50 ng/mL BMP4 (Fisher Scientific, 314BP01M) was added to the media in the induction channel. Thirty hours after the addition of BMP4, the devices were fixed in 4% PFA overnight. IF staining of the embryo model involved permeabilizing (PBS, 0.05% Triton-100) the stem cell models in the microfluidic device for 4 hours followed by incubation overnight in blocking solution (10% normal donkey serum in PBS). Devices were then incubated with primary antibodies at 4°C overnight, and then secondary antibodies overnight at 4°C. Finally, devices were treated with DAPI (Fisher Scientific, D1306) for one hour before imaging.

#### Microscopy

Confocal images of hPGCLCs within 3-D sectioned aggregates were examined on an LSM 880 (Carl Zeiss) with a Plan-Apochromat 20×/0.8 NA and a Plan-Apochromat 40×/1.4 NA M27 oil immersion objective at room temperature. Acquired images were processed using IMARIS 8.1 (Bitplane).

#### Image analysis

Images were processed using IMARIS 8.1 (Bitplane) microscopy image analysis software. To quantify the total number of NANOG/TFAP2C/PRDM1 triple-positive hPGCLCs that arose within the amniontic-ectoderm of the embryonic-like sac models, we quantified the total number of nuclei in this region using the spot detection function to detect DAPI. Only DAPI positive nuclei that overlapped with TFAP2C signals were counted, using the IMARIS spot function. Using the TFAP2C channel and the co-localization function in IMARIS we built co-localization channels for SOX17 and PRDM1 channels. From each Participant’s hiPSC subline, eight random embryonic-like sac images were assessed. Graphs were made using Prism 7 (Graph Pad) data analysis software and error bars represent the standard deviation. Image processing included using the mask function to detect and display only signal overlapping with DAPI-positive cell nuclei.

#### Processing aggregates for 10x genomics

Aggregates at day 4 of differentiation from MZT01, MZT02 MZT04, MZT05, MZT06 hiPSC sublines (15 sublines) were dissociated to single cells using Trypsin (0.05%). The single cell suspensions were resuspended in 1× PBS with 0.04% Bovine Serum Albumin (BSA), strained through a 40 μm strainer and counted using an automated cell counter (Thermo Fisher Scientific, Countess II). The cell concentration was adjusted to 800–1,200 cells/μl and immediately used for 10x Genomics. Following cell loading into the Chromium controller where individual cells were partitioned and barcoded, libraries were generated using the Chromium Single Cell 3′ Reagent Kit v2 according to the manufacturer’s instructions, and library fragment size distribution was determined using a BioAnalyzer instrument. Libraries were pooled and sequenced together in three separate sequencing runs (MZT01-F and MZT02-H; MZT05-F, MZT04-J, and MZT06-C; MZT01-E, MZT01-N, MZT02-D, MZT02-G, MZT04-D, MZT04-C, MZT06-A, MZT06-B) using Illumina Novaseq 6000 at an average depth of 500,000 million reads per sample.

#### Single-cell RNA-seq analysis of 10X genomics libraries

single-cell RNA-seq reads were aligned to the human hg38 genome assembly using 10x Genomics Cell ranger v.2.2. Expression matrices were generated by Cell Ranger. The generated cell-by-gene unique molecular identifier (UMI) count matrix was analyzed using Seurat R package v.2.3.4. Analysis was conducted on cells expressing at least 100 genes, and on genes expressed in at least 3 cells. Additional filtering involved including a maximum of 8,000 expressed genes per cell and excluding cells with >25% mitochondrial genes. The UMI counts were then normalized for total cell expression, multiplied by 10,000, and log-transformed. This unbiased approach yielded a total of 49,529 valid cells, including 2,562 germ cells and 46,962 somatic cells (Table S4). Seurat’s default method was used to identify highly variable genes and to scale data for regressing out variation from UMI and mitochondrial genes. Principal component analysis (PCA) was performed on the scaled data. The top 50 principal components were chosen for further analysis, including clustering to identify cell populations. UMAPs were calculated by RunUMAP function in Seurat package using top 50 PCs and min_dist = 0.30. Batch correction was conducted between the separate biological replicates using Seurat’s canonical correlation analysis procedure with default parameter. Briefly, this analysis identifies vectors with correlation between datasets and then aligns values along these vectors to reduce the variation from batch. The top 30 canonical correlation vectors were used further for clustering and UMAP visualization.

To compare the *in vitro* hPGCLC aggregates with *in vivo* PGCs, valid cells and UMIs were determined by UMI-tools to generate whitelist. Reads corresponding to valid barcodes were aligned to GRCh38 with STAR 2.7, and only uniquely mapped reads were kept for further analyses. Count matrices were generated by featureCounts v2.0.1 from the Subread R package, with UMIs info further appened to the alignment .bam file. Finally, the count matrix of all valid cells was generated with umi_tools count function.

Amnion-like cells within the aggregates derived from one hiPSC line from each human participant were identified based on expression of *IGFBP5* and *GABRP.* These cells were then isolated and compared to amnion-like cells previously identified in Zheng.^55^

Differential expressed gene (DEG) analysis between PGCLCs from each twin were calculated using a Wilcox Rank Sum Statistical test using the parameters of a ≥ 2-fold cut-off with genes expressed in >70% of PGCLCs. No statistically significant genes were identified.

#### SMARTseq analysis

Using the Carnegie Stage 7 (CS7) SMART-Seq raw data of annotated PGCs from Tyser,^53^ the SMARTseq raw reads were trimmed with cutadapt 1.18 and reads with length over 30 bp were aligned to GRCh38 with STAR 2.7, and only uniquely mapped reads were kept for further analyses. Count matrices were generated by featureCounts v2.0.1 from the Subread R package.

#### Principal component analysis (PCA)

Each scRNAseq library (week 13 hPGCs, Carneigie 7 hPGCs and MZT hPGCLCs) were normalized with edgeR R package to acquire a CPM (Count Per Million) matrix. Top 2000 variable genes were extracted to perform PCA with the prcomp() function in R.

#### Unsupervised hierarchical clustering (UHC)

The UHC was performed with scipy.cluster.hierarchy python function.

#### Whole genome sequencing

Raw sequencing reads were aligned to the Human reference genome GRCh37 using BWA-MEM v0.7.17. Duplicated reads were marked and removed using MarkDuplicates (Picard), base quality score recalibration was performed with BaseRecalibrator followed by PrintReads. Variates of each individual sample were called through GATK haplotypecaller v4.2.2.0 and filtered with criteria: 80 ≥ DP > 20, QUAL > 500, GQ > 50, MQ > 30 and MAF > 0.1, and the similarity coefficient (Jaccard index) were measured by pair-wise comparison to verify the genetic relationship of each biological sample. Two methods were applied and intersected to find high confidence variates in hiPSCs. Unique variates found by HaplotypeCaller were obtained from each hiPSC and fibroblast deduction. Secondly, each hiPSC and fibroblast comparison was verified though Strelka2 somatic mode, and the variates were filtered with criteria 80 ≥ DP > 20, MQ > 30 and MAF >0.1. Only variates found in both GATK and Strelka2 were counted. Libraries were pooled and sequenced together in three separate sequencing runs.

### Quantification and statistical analysis

All statistical analysis was performed using R data analysis software. A p value of <0.05 was considered statistically significant. For all experiments, data are expressed as the mean ± SD. Unpaired two-tailed t tests are used in all cases unless otherwise stated. Power analyses were performed before experiments to determine n values. Experimenter was blinded to fertility phenotype of the participants, HDFs, and hiPSC lines for the duration of the study. For each experiment the number of technical replicates, and the number of biological replicates in each group is reported. Statistical details of experiments can be found in figure legends.

## Data Availability

scRNA-seq data in this paper have been deposited at GEO Database: GSE181205 and are publicly available as of the date of publication. The accession number is also listed in the key resources table. Whole genome sequencing data have been deposited at NCBI BioProject Database: PRJNA759332 and are available upon the date of publication. Any additional information required to reanalyze the data reported in this paper is available from the lead contact upon request.

## Supplemental information

Document S1. Figures S1–S4 and Tables S1–S5

Document S2. Article plus supplemental information
