# Supplementary material for: In vitro germ cell induction from fertile and infertile monozygotic twin research participants
Source: Cell Rep Med. 2022 Oct 18;3(10):100782. doi: 10.1016/j.xcrm.2022.100782 (PMC9589117; doi:10.1016/j.xcrm.2022.100782)
Supplement: Document S1. Figures S1–S4 and Tables S1–S5 [file mmc1.pdf]

Cell Reports Medicine, Volume *3*

Supplemental information

In vitro germ cell induction from fertile and infertile monozygotic twin research participants

Erica C. Pandolfi, Fei-Man Hsu, Mark Duhon, Yi Zheng, Sierra Goldsmith, Jianping Fu, Sherman J. Silber, and Amander T. Clark

Supplementary Materials for

In Vitro Germ Cell Induction from Fertile and Infertile Monozygotic Twin Research Participants

Erica C. Pandolfi, Fei-Man Hsu, Mark Duhon, Yi Zheng, Sierra Goldsmith, Jianping Fu, Sherman J. Silber, Amander T. Clark

Correspondence to: clarka@ucla.edu

This PDF file includes:

Figures S1, S2, S3, and S4
Tables S1, S2, S3, S4 and S5

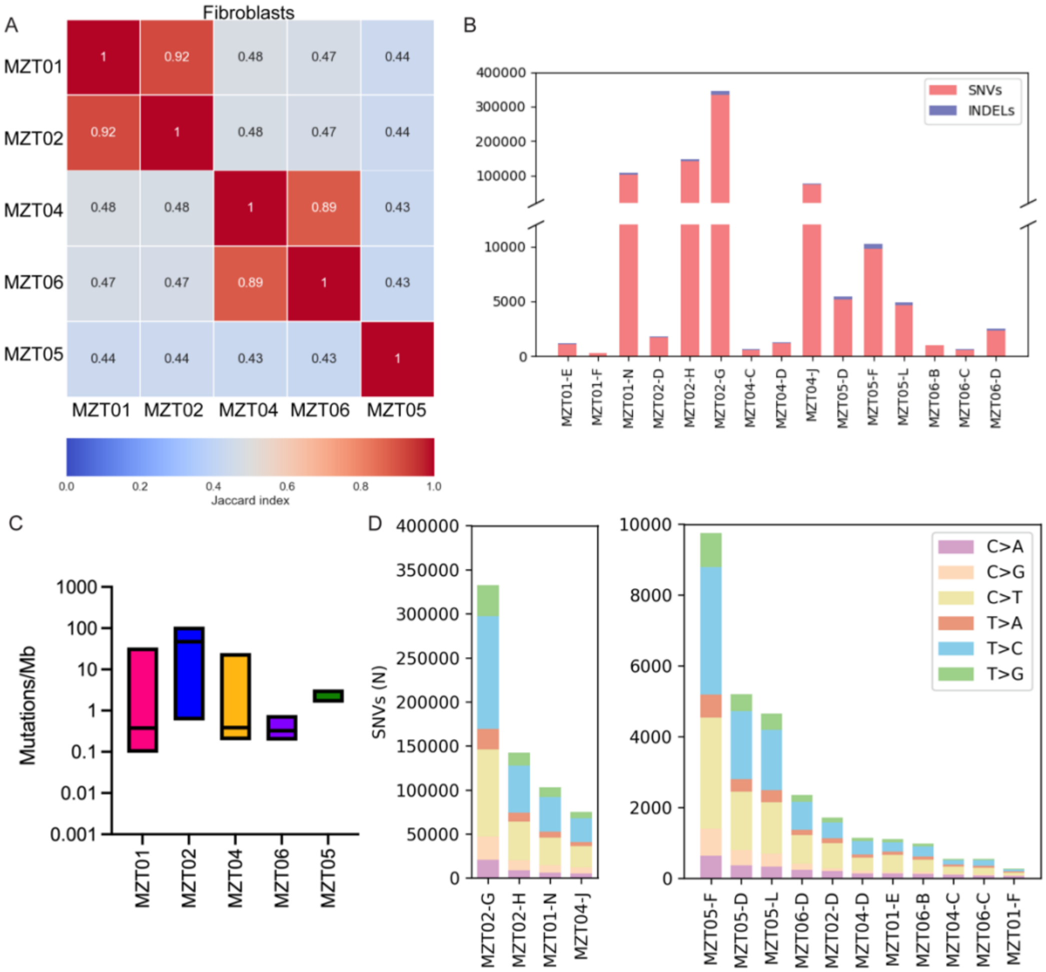

Figure S1. Whole Genome Sequencing (WGS) of twin HDFs and hiPSCs. Related to Figure 1. (A) Jaccard index comparison of HDFs to confirm relatedness of the twins. (B) Total number of acquired SNVs and INDELS in hiPSC sublines when compared to their HDF. (C) Average number of mutations per megabase of the three hiPSC sublines from each twin. (D) Proportions of SNV types from each hiPSC subline.

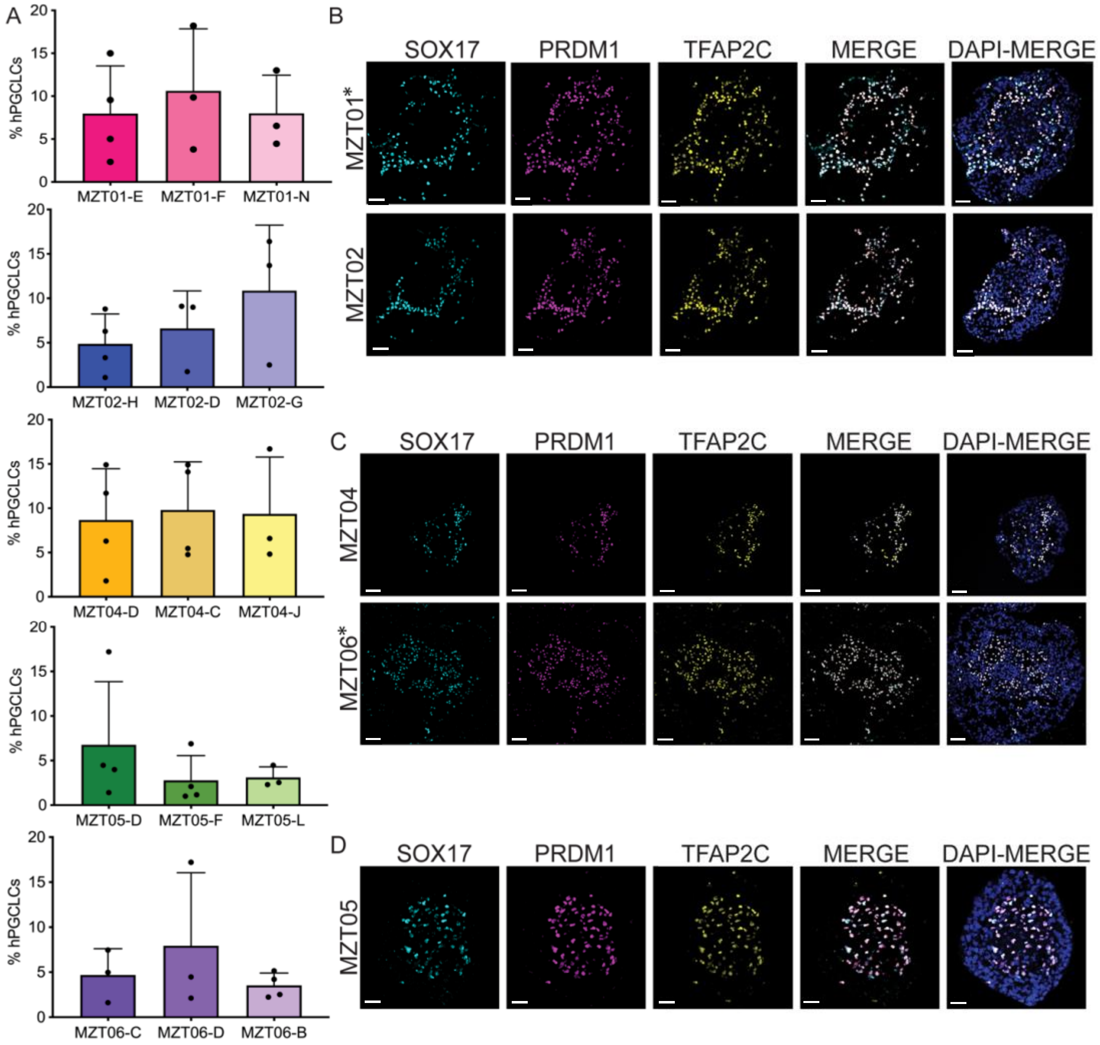

Figure S2. Characterization of hPGCLCs induced from hiPSCs. Related to Figure 2. (A) Percentage of hPGCLCs quantified by FACS from each participant's hiPSC sublines (ANOVA: MZT01-E, MZT01-F, and MZT01-N (F=0.22, p=0.81) ; MZT02-H, MZT02-D, and MZT02-G (F=1.24, p=0.57); MZT04-D, MZT04-C, and MZT04-J (F=0.197, p=0.825); MZT06-B, MZT06-C, and MZT06-D (F=0.78, p=0.42); MZT05-D, MZT05-L,and MZT05-F (F=0.86, p=0.46). n=3-4 independent experiments (biological replicates) for each subline. Representative images of hPGCLCs identified as being triple-positive for TFAP2C, PRDM1 and SOX17 in (B) MZT01 AND MZT02 (scale bar = 50μm), (C) MZT04 AND MZT06 (scale bar = 70μm), and (D) MZT05 (scale bar = 30μm).

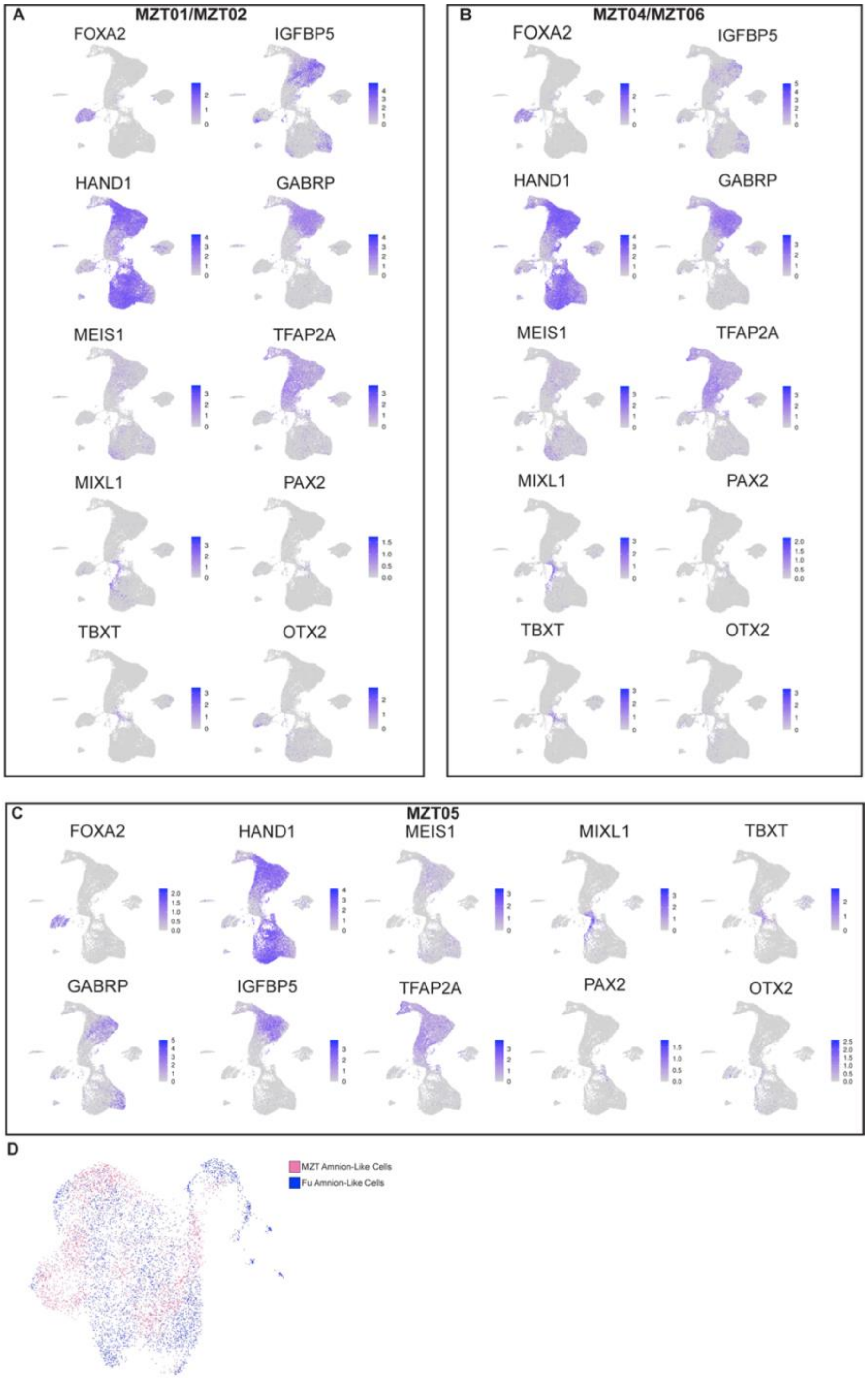

Figure S3. Expression of somatic lineage markers in the aggregates at day 4. Related to Figure 3. Each hiPSC subline was evaluated for expression of somatic lineage markers (n=3 for each participant). (A) Twin pair MZT01 and MZT02. (B) Twin pair MZT04 and MZT06. (C) Twin MZT05. (D) Comparison of amnion-like cells within the MZT aggregates to amnion-like cells identified in Zheng et al. [55].

Figure S4.

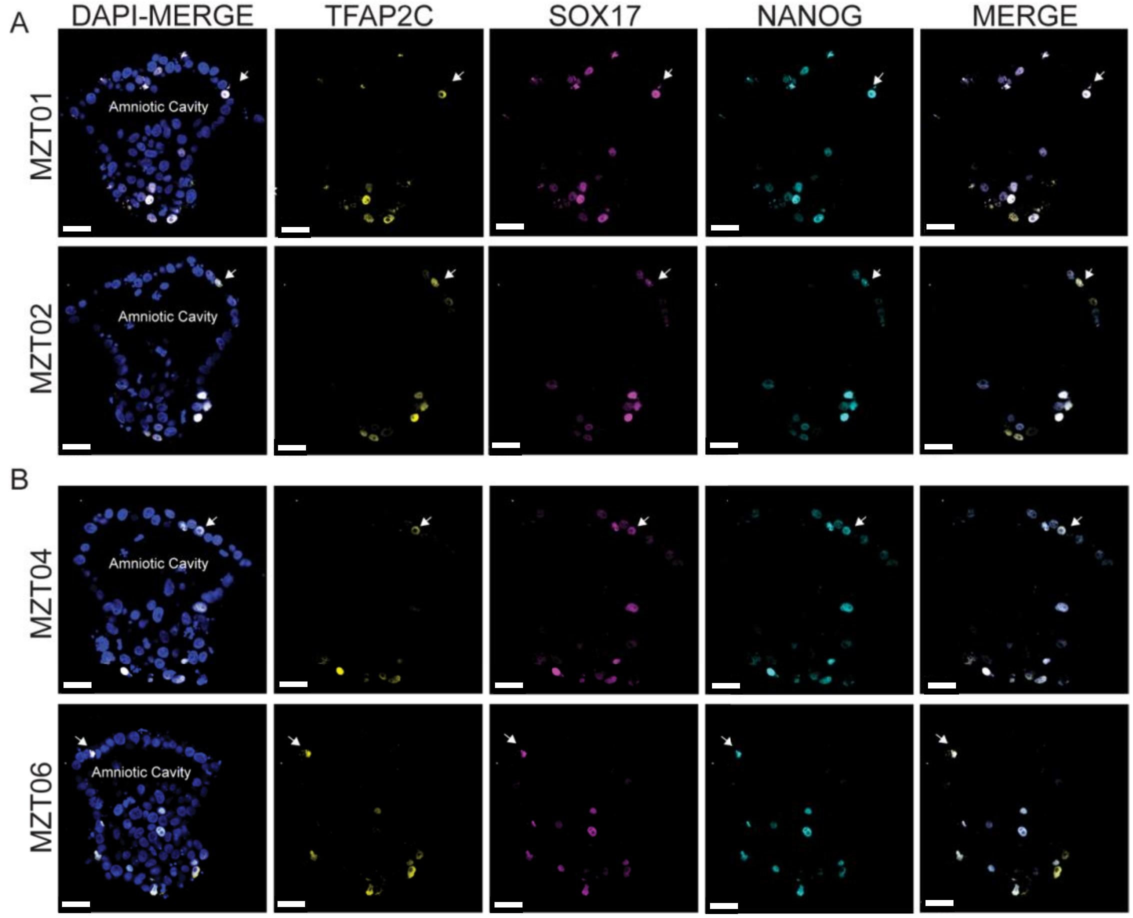

Figure S4. Presence of hPGCLCs in the embryo model of the amniotic sac. Related to Figure 4. hPGCLCs (cells triple-positive for TFAP2C, NANOG, and SOX17) in amniotic ectoderm layer (Arrows) and pre-primitive streak EPI layer of the embryo models generated from sublines MZT01-E and MZT02-G (A), and sublines MZT04-C and MZT06-C (B). Scale bars = 30μm)

Table S1.

Sample ID	Diagnosis	Karyotype	Age of Menopause	Age at Biopsy	Ethnicity	Type of MZ Twin	Successful Pregnancy w/out Intervention?	Fertility Intervention	Successful Pregnancy w/ Intervention?
MZT01	POI	Normal	25	39	White	MC, MA	No	Ovarian Transfer	Yes, 2 babies
MZT02	Normal	Normal	Unknown	39	White	MC, MA	Yes	N/A	N/A
MZT03	POI	Abnormal	31	47	Asian	Unknown	No	Ovarian Transfer	Yes,1 baby
MZT04	Normal	Normal	Unknown	52	White	MC, MA	Yes	N/A	N/A
MZT05	Normal	Normal	Unknown	47	Asian	Unknown	Unknown	No	Unknown
MZT06	POI	Normal	22	53	White	MC, MA	No	Ovarian Transfer	Yes,3 babies

Table S1: Participant demographics and health information. Related to Figure 1. *MZ = Monozygotic MC = Monochorionic, MA =Monoamniotic. MA twins are also MZ and MC.

Table S2.

Sample		P	Total Reads	Mapped Reads (%)	Coverage (X)	SNV	INDEL
HDF	MZT01	5	1,597,970,064	1,585,867,860 (99.2%)	75.8	N/A	N/A
	MZT02	5	1,495,400,650	1,483,604,495 (99.2%)	70.9	N/A	N/A
	MZT04	3	994,588,972	981,256,126 (98.7%)	46.9	N/A	N/A
	MZT05	1	1,388,686,994	1,379,198,676 (99.3%)	65.9	N/A	N/A
	MZT06	1	934,494,388	925,001,799 (99.0%)	44.2	N/A	N/A
hiPSC	MZT01-E	11	876,733,766	779,139,165 (88.9%)	37.3	1107	82
	MZT01-F	11	990,866,782	935,821,423 (94.4%)	37.3	276	22
	MZT01-N	11	947,476,403	672,252,714 (71.0%)	32.1	103050	4082
	MZT02-D	5	974,094,172	860,592,337 (88.3%)	41.1	1720	100
	MZT02-G	5	1,267,010,004	781,345,938 (61.7%)	37.4	332530	11349
	MZT02-H	18	830,386,870	539,715,756 (65.0%)	25.8	142265	6127
	MZT04-C	11	790,487,368	728,783,903 (92.2%)	34.8	555	46
	MZT04-D	22	901,928,786	796,819,387 (88.3%)	38.1	1147	70
	MZT04-J	8	872,429,436	618,537,285 (70.9%)	29.6	75308	2832
	MZT05-D	15	889,227,700	737,606,377 (82.9%)	35.3	5208	230
	MZT05-F	16	1,057,621,294	880,600,229 (83.3%)	42.1	9757	469
	MZT05-L	16	874,530,626	725,062,322 (82.9%)	34.7	4653	240
	MZT06-B	6	758,779,888	674,253,304 (88.9%)	32.2	979	40
	MZT06-C	6	893,201,272	820,387,371 (91.8%)	39.2	552	38
	MZT06-D	6	875,043,360	758,886,391 (86.7%)	36.3	2358	96

Table S2. Sequencing summary of the whole genome sequencing experiments. Related to Figure 1 and Figure S1. p= passage. N/A = Not applicable.

Table S3.

Genes Queried	Mutation in protein coding region?
HFM1	No
CYP17A1	No
FSHB	No
EIF2B1	No
CYP19A1	No
POLG	No
PMM2	No
CLPP	No
FSHR	No
MCM8	No
LARS2	No
FOXL2	No
HSD17B4	No
MSH5	No
AARS2	No
MCM9	No
STAG3	No
NOBOX	No
GALT	No
NR5A1	No
SOHLH1	No
BMP15	No

Table S3. POI candidate genes queried for presence of genomic discrepancy. Related to Figure 1 and Figure S1.

Table S4.

Cell line	Raw reads	Sample Details	Read with valid barcodes	Uniquely mapped reads	Mapping Rate (%)	Valid cells
MZT01-E	307,389,339	Aggregates at day 4	224,708,530	187,291,630	83.35	2,240
MZT01-F	293,116,876	Aggregates at day 4	250,723,753	202,784,174	80.88	6,698
MZT01-N	246,219,391	Aggregates at day 4	211,367,881	179,181,705	84.77	10,544
MZT02-D	541,854,568	Aggregates at day 4	359,577,689	297,420,458	82.71	6,297
MZT02-G	303,344,042	Aggregates at day 4	135,618,433	111,538,505	82.24	3,025
MZT02-H	156,488,911	Aggregates at day 4	129,855,114	104,110,150	80.17	5,908
MZT04-C	530,077,480	Aggregates at day 4	406,628,621	339,264,406	83.43	7,807
MZT04-D	345,491,697	Aggregates at day 4	252,355,587	214,913,231	85.16	3,015
MZT04-J	283,916,773	Aggregates at day 4	232,520,453	176,123,670	75.75	7,536
MZT05-D	421,387,687	Aggregates at day 4	355,842,847	277,540,338	78.00	9,453
MZT05-F	369,117,089	Aggregates at day 4	287,623,816	251,110,341	87.31	5,256
MZT05-L	322,110,849	Aggregates at day 4	146,865,388	122,199,397	83.21	3,172
MZT06-A	165,543,605	Aggregates at day 4	140,893,958	118,358,651	84.01	11,148
MZT06-B	166,702,330	Aggregates at day 4	140,641,454	118,626,196	84.35	9,726
MZT06-C	348,215,615	Aggregates at day 4	204,804,753	166,183,202	81.14	6,026

Table S5. Sequencing summary of the 10X Genomics scRNA-seq experiments. Related to Figure 3.
